# Interstitial oxygen as a source of *p*-type conductivity in hexagonal manganites

**DOI:** 10.1038/ncomms13745

**Published:** 2016-12-07

**Authors:** Sandra H. Skjærvø, Espen T. Wefring, Silje K. Nesdal, Nikolai H. Gaukås, Gerhard H. Olsen, Julia Glaum, Thomas Tybell, Sverre M. Selbach

**Affiliations:** 1 Department of Materials Science and Engineering, NTNU Norwegian University of Science and Technology, NO-7491 Trondheim, Norway; 2 Department of Electronics and Telecommunications, NTNU Norwegian University of Science and Technology, NO-7491 Trondheim, Norway

## Abstract

Hexagonal manganites, h-*R*MnO_3_ (*R*=Sc, Y, Ho–Lu), have been intensively studied for their multiferroic properties, magnetoelectric coupling, topological defects and electrically conducting domain walls. Although point defects strongly affect the conductivity of transition metal oxides, the defect chemistry of h-*R*MnO_3_ has received little attention. We use a combination of experiments and first principles electronic structure calculations to elucidate the effect of interstitial oxygen anions, O_i_, on the electrical and structural properties of h-YMnO_3_. Enthalpy stabilized interstitial oxygen anions are shown to be the main source of *p*-type electronic conductivity, without reducing the spontaneous ferroelectric polarization. A low energy barrier interstitialcy mechanism is inferred from Density Functional Theory calculations to be the microscopic migration path of O_i_. Since the O_i_ content governs the concentration of charge carrier holes, controlling the thermal and atmospheric history provides a simple and fully reversible way of tuning the electrical properties of h-*R*MnO_3_.

## Abstract (web summary)

Hexagonal manganites are multiferroic materials that can exhibit magnetoelectric coupling and electrically conducting domain walls. Here, the authors produce yttrium manganite with *p*-type conductivity, useful for devices, by using excess oxygen to introduce point defects in the crystal lattice.

Point defects are imperative to the functional properties of oxides used in electrochemical devices like solid oxide fuel cells, batteries and memristors^1^,^2^,^3^. In contrast, point defects in general have a detrimental effect on physical properties of oxides for electronics, such as for example fatigue and domain wall pinning in ferroelectrics^4^. As components are made smaller, the available length for point defects in materials to diffuse is made shorter. This reduces the time required for the point defect to diffuse through the samples compared with bulk materials, where they effectively freeze in^5^. Understanding point defects in functional oxides thus becomes ever more important with decreasing component dimensions^6^,^7^.

Rare earth ternary manganites with *R*MnO_3_ stoichiometry are stable in the hexagonal manganite structure with space group *P*6_3_*cm* (185) for *R*=Sc, Y and Ho–Lu. The structure consists of layers of five-coordinated Mn^3+^ corner-sharing trigonal bipyramids separated by layers of Y^3+^ in the *ab*-plane. The MnO_5_ bipyramids are tilted in a pattern of trimers, while the Y^3+^ are displaced in opposite directions along the polar *c*-axis^8^. A subtle shift of the trigonal bipyramid layer with respect to the Y^3+^ layer, caused by an improper ferroelectric transition at 1,250 K, is the origin of the ferroelectric polarization^9^. The Mn^3+^ sublattice has a frustrated non-collinear antiferromagnetic order on a trigonal lattice, with a Néel temperature of 75 K (ref. 10).

Compared with the ternary ABO_3_ perovskite oxides, the defect chemistry of h-*R*MnO_3_ has received little attention. Oxygen vacancies in YMnO_3_ have been studied^11^,^12^,^13^, and large oxygen *excess* has been reported for h-*R*MnO_3+*δ*_ (*R*=Y, Dy) with *δ* up to 0.35 for *R*=Dy^14^. High levels of excess oxygen can cause development of secondary oxidized phases, which are easily detected experimentally^15^. However, structural effects, energetics and kinetics of point defects in low concentrations have not been addressed. Excess oxygen is well known in perovskite manganites such as LaMnO_3+*δ*_. Since the perovskite structure is too close-packed for interstitial oxygen anions, excess oxygen is accommodated by cation vacancies; La_1−*δ*/3_Mn_1−*δ*/3_O_3_ (ref. 16). Oxidation of perovskite LaMnO_3_ thus requires cation diffusion, which demands higher temperatures than anion diffusion.

Diffusion of oxygen in bulk YMnO_3_ has been observed below 200 °C (ref. 14), making cation vacancy compensation highly unlikely. The layered h-*R*MnO_3_ structure is however ∼11% less dense than the corresponding orthorhombic perovskite structure, suggesting the possibility of interstitial oxygen. This is further supported by the observation that crystallization, which requires cation diffusion, of amorphous YMnO_3_ only occurs above 800 °C (ref. 17).

Here we investigate how excess oxygen in the form of highly mobile interstitial anions is accommodated in the hexagonal manganite structure, and demonstrate that this enthalpy stabilized point defect gives rise to the observed *p*-type conductivity in YMnO_3_. This shows the importance of controlling the material's thermo-atmospheric history and opens a new avenue for tuning the physical properties of hexagonal manganites.

## Results

### Oxygen stoichiometry and electrical conductivity

**Figure 1:**
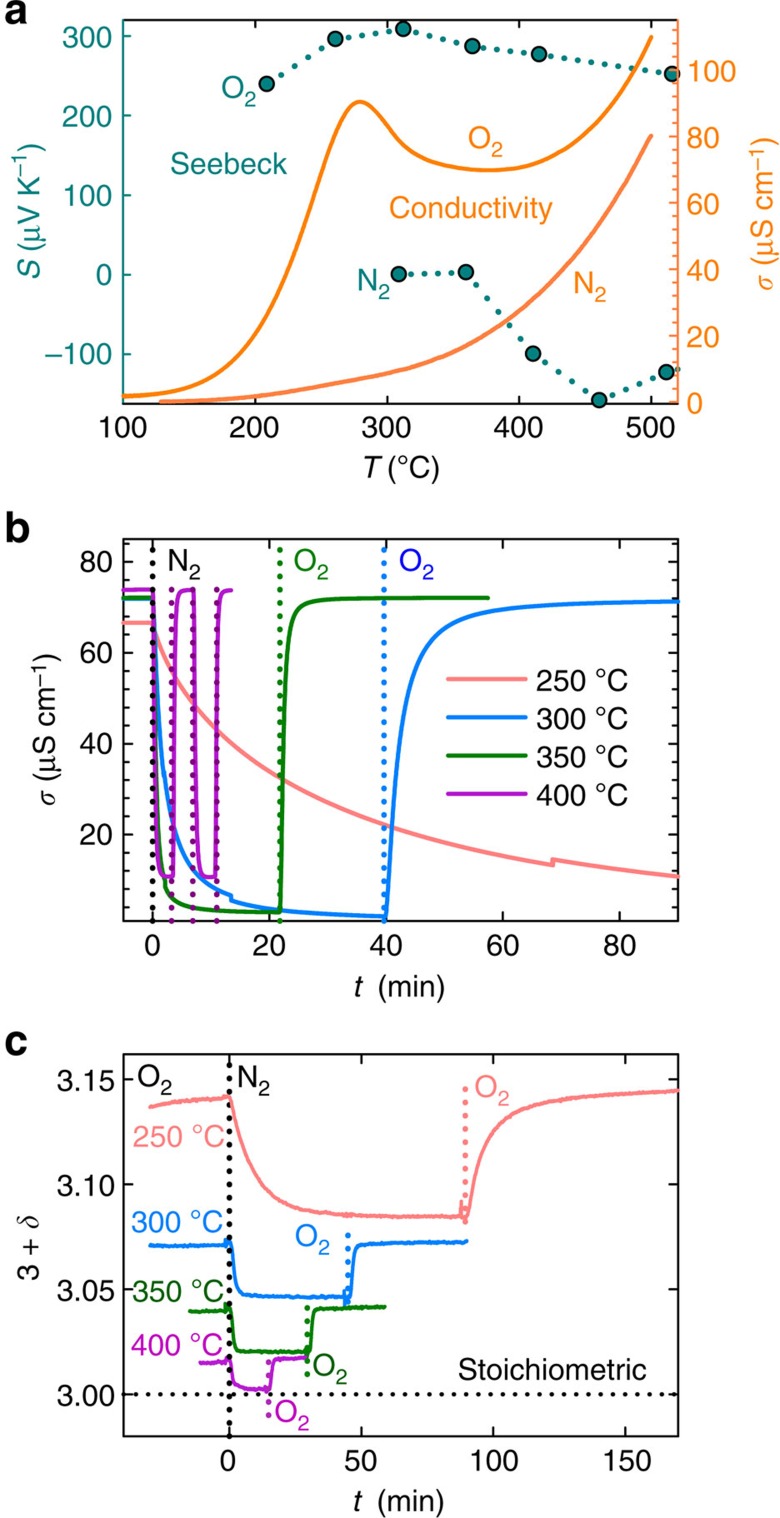
*p*-type conductivity and excess oxygen. (**a**) Seebeck coefficient, *S*, and DC electrical conductivity, *σ*, in O_2_ and N_2_ atmosphere of a porous polycrystal of YMnO_3_, as a function of temperature, *T*. (**b**) DC electrical conductivity as a function of time, *t*, of a porous polycrystal upon switching atmosphere (indicated by dotted lines) between flowing O_2_ and N_2_. The atmosphere is switched twice between O_2_ and N_2_ at 400 °C. (**c**) Oxygen stoichiometry, 3+*δ*, as a function of time, *t*, upon switching atmosphere measured by thermogravimetry for YMnO_3_ nanoparticles (*d*_XRD_=49±4 nm).

First we consider the effect of thermal and atmospheric history on the conductivity and oxygen stoichiometry of the prototypical hexagonal manganite YMnO_3_. Thermoelectric power for a porous polycrystalline YMnO_3_ bar was measured in O_2_ and N_2_ atmosphere, Fig. 1a. The Seebeck coefficient is positive for all temperatures in O_2_ atmosphere, implying that *p*-type electronic conduction dominates, and a maximum is observed at 300 °C. In N_2_ atmosphere, the Seebeck coefficient is negative, implying conduction by electrons as majority charge carriers. The DC electrical conductivity of YMnO_3_ in O_2_ increases exponentially on heating from 100 °C, as expected for a semiconducting oxide. However, the conductivity goes through a maximum value at 270 °C, then decreases and stays relatively constant up to 420 °C, from which the conductivity again increases exponentially. In N_2_ atmosphere, the DC conductivity follows an exponential trend on heating, as expected for a semiconductor.

The observations in Fig. 1a is consistent with the chemical defect reaction:


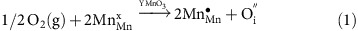


where 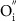 is interstitial O^2−^, and 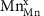 and 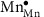 depicts Mn^3+^ and Mn^4+^ on Mn lattice sites, respectively. Tetravalent Mn in oxidized YMnO_3_ is Mn with localized electron holes: 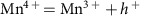. Holes as the majority charge carrier is consistent with the positive sign of the Seebeck coefficient. The mobility of negatively charged interstitial O^2−^ is expected to be orders of magnitude smaller than for holes, and this is addressed further below.

The peculiar thermal evolution of the electrical conductivity can be explained from the maximum oxygen stoichiometry observed at about 250 °C (ref. 14), which coincides with a maximum in electrical conductivity. The deviation in conductivity from the exponential behaviour of a semiconductor stems from the different temperature dependence of the concentration and mobility of holes. While the mobility of holes increases with temperature, this is counterbalanced by the loss of oxygen and charge compensating holes, above 250 °C, as we will show further below. The atypical thermal evolution of the electrical properties of YMnO_3_ in this temperature range has previously been attributed to the filling of oxygen vacancies^18^,^19^,^20^, but charge compensating electrons would give a negative Seebeck coefficient.

DC conductivity measurements were then performed on isothermal change of atmospheres between O_2_ and N_2_. When the partial pressure of oxygen, *p*O_2_, is reduced by switching from O_2_ to N_2_ atmosphere, the oxygen excess decreases according to equation (1) and a relaxation towards a lower conductivity is observed in Fig. 1b. The initial conductivity is regained after changing the atmosphere back to O_2_, demonstrating the reversibility of reaction (1). The regained conductivity is similar for all isotherms, owing to the opposing effects of decreasing charge carrier mobility and increasing concentration as the temperature is reduced. The relaxation time before equilibrium is reached is highly temperature dependent, as expected for diffusion of oxygen into the lattice.

AC conductivity measurements were performed on samples annealed in flowing O_2_ and N_2_, respectively, before measurement (Supplementary Fig. 1). The conductivity of the O_2_ annealed sample shows the characteristic maximum at 280 °C on heating, resembling the maximum observed in Fig. 1a. The conductivity measured on cooling is lower compared with the initial heating cycle reflecting the loss of O_i_ that occurred at higher temperatures. The N_2_ annealed sample exhibits a smooth increase of the conductivity with temperature. Subsequent cooling leads to higher conductivity in accordance with the reversibility of the O_i_ incorporation process.

The reversibility of reaction (1) is also evidenced by the oxygen stoichiometry when switching between O_2_ and N_2_ atmospheres, Fig. 1c. As the length scale of the system is imperative to diffusion controlled processes^5^, nanoparticles with an average crystallite size of *d*_XRD_=49±4 nm (ref. 17) were chosen for studying thermogravimetric relaxation on switching atmosphere at a shorter time-scale than in bulk material. With increasing temperature the oxygen stoichiometry equilibrates faster. The rapid change in oxygen stoichiometry in YMnO_3_ nanoparticles illustrates the increasing importance of thermal and atmospheric history with decreasing system size. The isothermal response of the electrical conductivity (Fig. 1b) and the oxygen stoichiometry (Fig. 1c) on change in *p*O_2_ is explained by reaction (1): oxygen hyperstoichiometry governs the electrical conductivity of h-*R*MnO_3+*δ*_.

### Position of interstitial oxygen and structural effects

**Figure 2:**
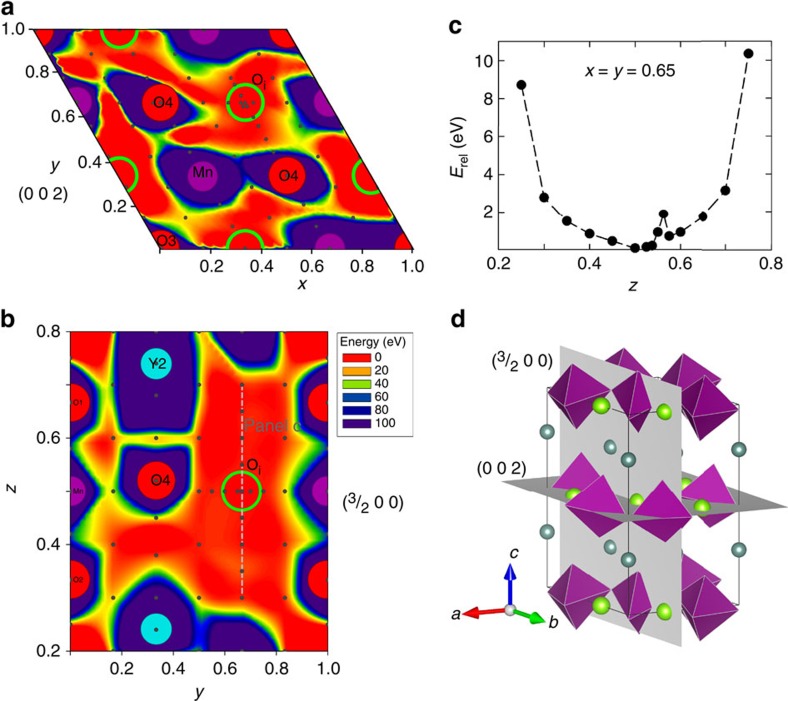
Position of interstitial oxygen. Potential energy surfaces (PES) showing the energy landscape for the position of interstitial oxygen in a neutral YMnO_3_ unit cell in (**a**) the (002) plane and (**b**) the (^3^/_2_00) plane. (**c**) Relative energy along the *z* direction for *x*=*y*=0.65 indicated by a light grey line in panel **b**. The anomaly at *z*=0.56 stems from that the PES mapping was done with static calculations. (**d**) Crystal structure of YMnO_3_ showing purple MnO_5_ polyhedra, turquoise Y atoms, and green spheres marking the six equivalent stable positions for O_i_ in the unit cell along with the two crystal planes in (**a**),(**b**).

The low temperature at which YMnO_3_ exchanges oxygen with the atmosphere makes cation vacancies and diffusion unrealistic, and the positive Seebeck coefficient from mobile holes points to interstitial oxygen anions, O_i_, as the dominating point defect. High-temperature X-ray diffraction measurements of nanocrystalline YMnO_3_ show that interstitial oxygen is incorporated into the lattice, causing anisotropic chemical expansion (Supplementary Fig. 2). We now turn our attention to the position of interstitial oxygen in YMnO_3_. Potential energy surfaces (PES) were determined by mapping the energy landscape of the O_i_ position in several lattice planes by static Density Functional Theory (DFT) calculations (See details in Supplementary Fig. 3 and Supplementary Note 1). For illustration, the PES of O_i_ in the (002) (**a**) and (^3^/_2_00) (**b**) planes are included in Fig. 2 along wth a unit cell with the corresponding lattice planes (**d**). The relative energy of O_i_ along the grey line in Fig. 2b is shown in Fig. 2c. The most stable positions of O_i_ was found to be between three Mn in the Mn–O planes at z=0 and ^1^/_2_, resulting in six equivalent possible sites for O_i_ in the *P*6_3_*cm* unit cell, (^1^/_3_, ^1^/_3_, 0), (^2^/_3_, 0, 0), (0, ^2^/_3_, 0), (^2^/_3_, ^2^/_3_, ^1^/_2_), (^1^/_3_, 0, ^1^/_2_) and (0, ^1^/_3_, ^1^/_2_), as illustrated by green circles in panels **a** and **b**.

Experimental observations of a maximum *δ* of ∼0.35 (refs 14, 15) can be rationalized from our DFT simulations. If two out of the six possible stable interstitial sites in the unit cell are occupied, a hypothetical fully oxidized structure of YMnO_3_ would have a chemical formula of YMnO_3.33_ or Y_3_Mn_3_O_10_. This will result in one O_i_ and two out of three oxidized Mn in each Mn–O layer of the 30 atom unit cell. Accommodation of more than two O_i_ per unit cell would require charge compensation across layers or further oxidation of Mn^4+^ to Mn^5+^ within the same layer.

**Figure 3:**
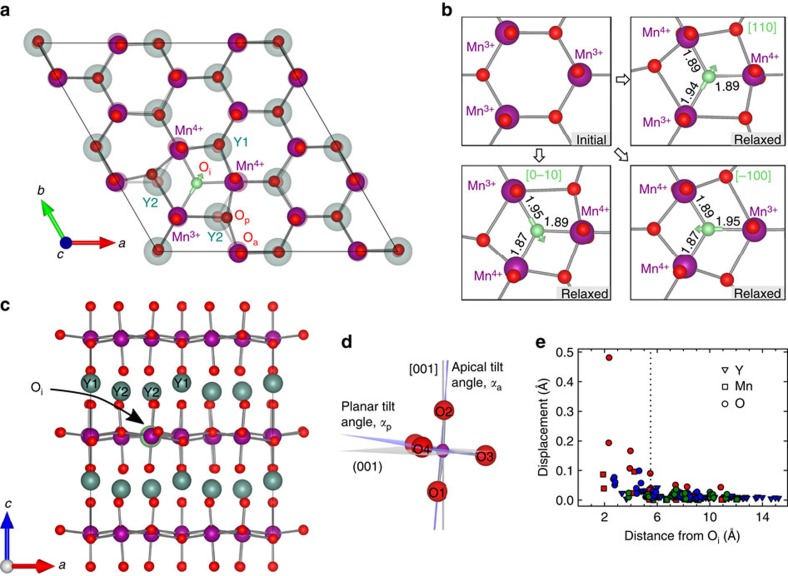
Structural distortions and screening length. (**a**) Relaxed geometry of the (002) plane of a 2 × 2 × 1 supercell of YMnO_3_ with the perfect structure faded in the background for comparison. Y atoms above the (002) Mn–O plane are projected onto the plane. (**b**) Unrelaxed structure in upper left panel compared with relaxed structures around O_i_ in the three triple-well positions around the ^2^/_3_, ^2^/_3_, ^1^/_2_ position. The green arrows on O_i_ indicate the displacement towards the two Mn^4+^. Bond lengths between O_i_ and Mn are given in Å. (**c**) YMnO_3_ with O_i_ (green sphere in the (002) Mn–O layer) viewed along the **b**-direction. (**d**) Apical and planar tilting angles of the trigonal bipyramids. O1 and O2; and O3 and O4 being apical and planar oxygens, respectively. (**e**) Displacements of ions with respect to the perfect structure as a function of distance from O_i_. The red symbols represent atoms in the same Mn–O layer as O_i_ is positioned, the blue symbols represent the apical oxygens and the yttrium atoms closest to the Mn–O layer with O_i_, and the green symbols show atoms in the adjacent Mn–O layers. The structural screening length is indicated by a vertical dotted line.

The in-plane distances between O_i_ and Mn after structural relaxation depicted in Fig. 3a,b show that there are two shorter and one longer Mn–O_i_ bond. O_i_ is displaced towards two *d*^3^ Mn^4+^ ions (with a calculated magnetic moment of 3.06 *μ*_B_), and away from one *d*^4^ Mn^3+^ ion (3.74 *μ*_B_). The localization of holes on the two oxidized Mn ions gives three possible configurations of O_i_ and its three surrounding Mn ions. The most stable positions of O_i_ are triple wells due to asymmetric electrostatic attraction: O_i_ with formal charge −2 has shorter bonds to the two Mn^4+^ than to the single Mn^3+^. Details and discussion about the energetic asymmetry of this triple well and the effect of O_i_ on the magnetism can be found in Supplementary Figs 4 and 5 and Supplementary Note 2.

Localization of holes on Mn, and the resulting *p*-type polaronic conduction, stems from electrostatic attraction between positive holes and negative interstitial anions. In contrast, hole doping by substituting Y^3+^ with Ca^2+^ gives holes in Bloch states, while electron doping by Zr^4+^ for Y^3+^ substitution gives a polaronic state^21^. The energy barrier for moving the holes associated with O_i_ between different Mn^4+^ pairs is 0.29 eV from nudged-elastic band (NEB) calculations (See details in Supplementary Fig. 6 and Supplementary Note 3). Our calculated energy barrier is lower than the reported activation energy of 0.38–0.50 eV for polaron hopping in YMnO_3_ (refs 22, 23, 24).

Interstitial oxygen causes only subtle local structural distortions, as evident from the excerpt of the (002) plane of YMnO_3_ in Fig. 3a. The relaxed atomic positions are shown in the foreground, while the atoms in a perfect crystal are included in faded colours in the background for comparison. The Y sublattice is virtually unaffected by the introduction of O_i_ in the Mn–O layer, as shown in Fig. 3c. The local displacements of ions occur mainly within the same layer of trigonal MnO_5_ bipyramids as O_i_ is situated, and the distortions decrease rapidly with increasing distance from O_i_. The apical tilting angle, defined in Fig. 3d, of the three bipyramids surrounding O_i_ is reduced from 8.7° in the perfect crystal to 6.4° for Mn^4+^ bipyramids and 4.7° for Mn^3+^ bipyramids, respectively.

The local structural distortions caused by the introduction of O_i_ can be quantified by the displacements of ions relative to the perfect *P*6_3_*cm* structure as a function of the distance from the O_i_, as illustrated in Fig. 3e. Displacements of Mn and planar oxygen closest to O_i_ are substantial, apical oxygen in the nearest bipyramids are also displaced, while Y even close to O_i_ are less affected. The structural screening length of the O_i_ point defect is defined here as the distance away from O_i_ where the displacement is less than 0.1 Å relative to the perfect structure. A structural screening length can thus be estimated to 5.5 Å, corresponding to the second coordination shell of O_i_, and is shown by a dotted line in Fig. 3e. Local charge compensation of O_i_ by two Mn^4+^ and polyhedral tilting mitigates the effect of O_i_ on the lattice. The short structural screening length implies that direct experimental detection of O_i_ is challenging.

The calculations also show that the non-collinear triangular magnetic order is only subtly affected by O_i_ (Supplementary Fig. 5). At the relevant temperatures for O_i_ transport, the material is paramagnetic and the properties are therefore not expected to be significantly affected by magnetic order. The relaxed structure is not affected by substituting the true non-collinear magnetic ground state structure with a synthetic collinear magnetic order.

### Energetics of O_i_

**Figure 4:**
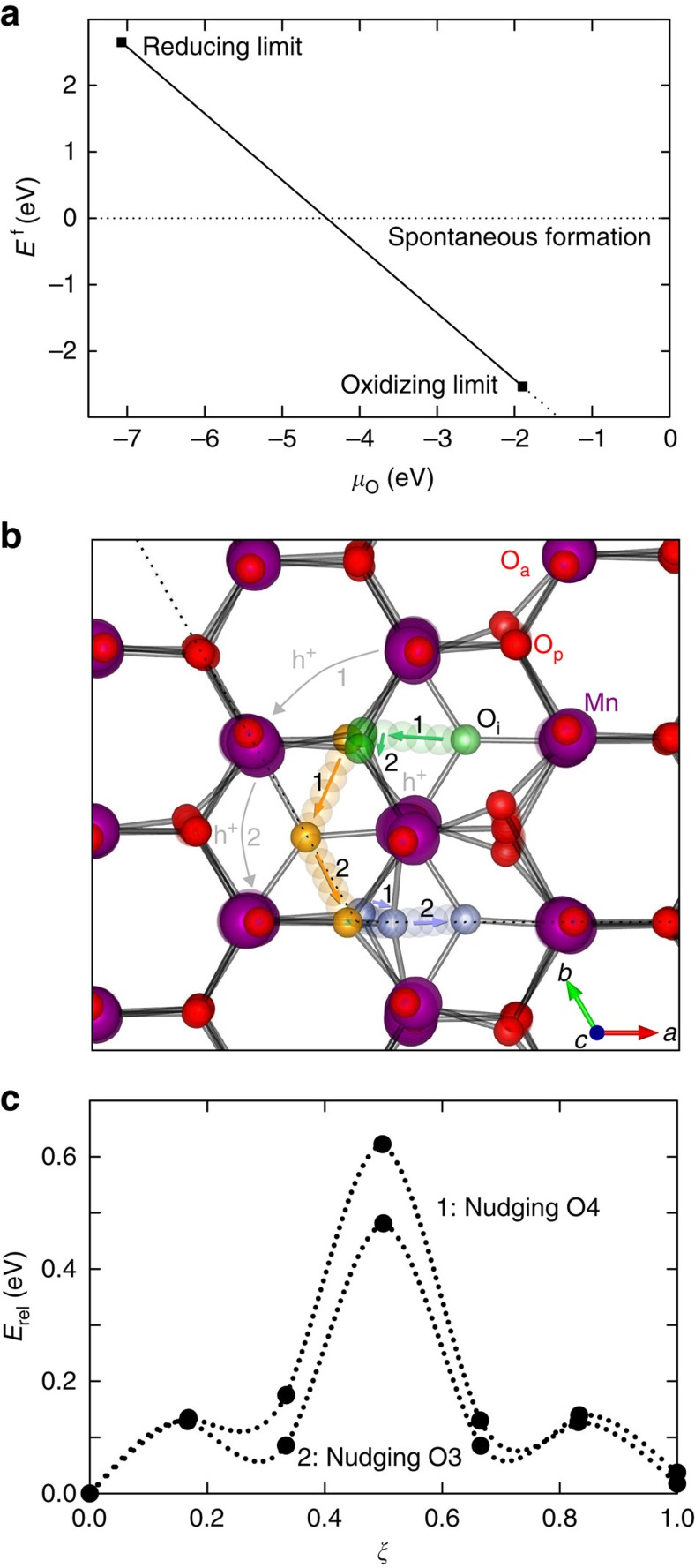
Energetics and kinetics. (**a**) Defect formation energy of O_i_ in a 2x2x1 supercell of YMnO_3_ as a function of chemical potential of oxygen. (**b**) Subsequent migration paths of O_i_ (green, then yellow and finally blue) through an interstitialcy mechanism nudging first a planar lattice oxygen at an O4 site (path 1) and then a planar lattice oxygen at an O3 site (path 2). The hopping of holes (h^+^) between Mn sites are shown by grey arrows. (**c**) The migration energy barriers for path 1 (nudging an O4) and path 2 (nudging an O3) as functions of the relative reaction coordinate, *ξ*.

An essential thermodynamic quantity for point defects is the energy of formation. Using the formalism of Zhang *et al*.^25^ for neutral cells we can define the energy of formation, *E*^f^, for interstitial oxygen as:


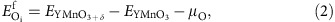


where 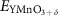 and 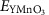 are the total energies of oxidized and stoichiometric YMnO_3_, respectively, and *μ*_O_ is the chemical potential of oxygen. The formation energy for O_i_ in YMnO_3_ as a function of the chemical potential of oxygen is shown in Fig. 4a (See Supplementary Fig. 7 and Supplementary Methods for details on the thermodynamic stability region for bulk YMnO_3_). For 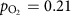, corresponding to air, the chemical potential of oxygen yields a negative formation energy at temperatures up to ∼900 °C, which is well above the temperature were O_i_ becomes entropy destabilized, as seen from the experimental measurements in Fig. 1. However, it must be pointed out that the formation enthalpy for O_i_ is calculated in the dilute limit, while a finite concentration of O_i_ is necessary to detect mass changes by thermogravimetry. The consumption of the gaseous species O_2_ (g) in reaction (1) means that the entropy of this reaction is negative, hence the entropy contribution to the Gibbs free energy of (1) is positive. Given the negative enthalpy of reaction (1) from DFT, the Gibbs' free energy of (1) becomes less negative with increasing temperature, gradually shifting (1) towards the left hand side where O_i_ leaves the lattice to form O_2_ molecules. The equilibrium concentration of O_i_ hence decreases with increasing temperature, in line with the results in Fig. 1, but in contrast to entropy stabilized oxygen vacancies which have a positive enthalpy of formation under conditions where the oxide is stable. In analogy to brownmillerite AB_2_O_5_ and perovskite ABO_3_, O_i_ in YMnO_3_ could be considered filled oxygen vacancies in the hypothetical compound Y_3_Mn_3_O_10_. With Y_3_Mn_3_O_10_ as the reference state, vacant O_i_ sites would be oxygen vacancies, and thus conform to conventional point defect thermodynamics with positive enthalpies and entropies of formation. We note that the enthalpy of formation for O_i_ is expected to decrease progressively with increasing oxygen excess *δ* due to O_i_–O_i_ repulsion and a gradual loss of the driving force for oxidation.

### Migration of O_i_

**Figure 5:**
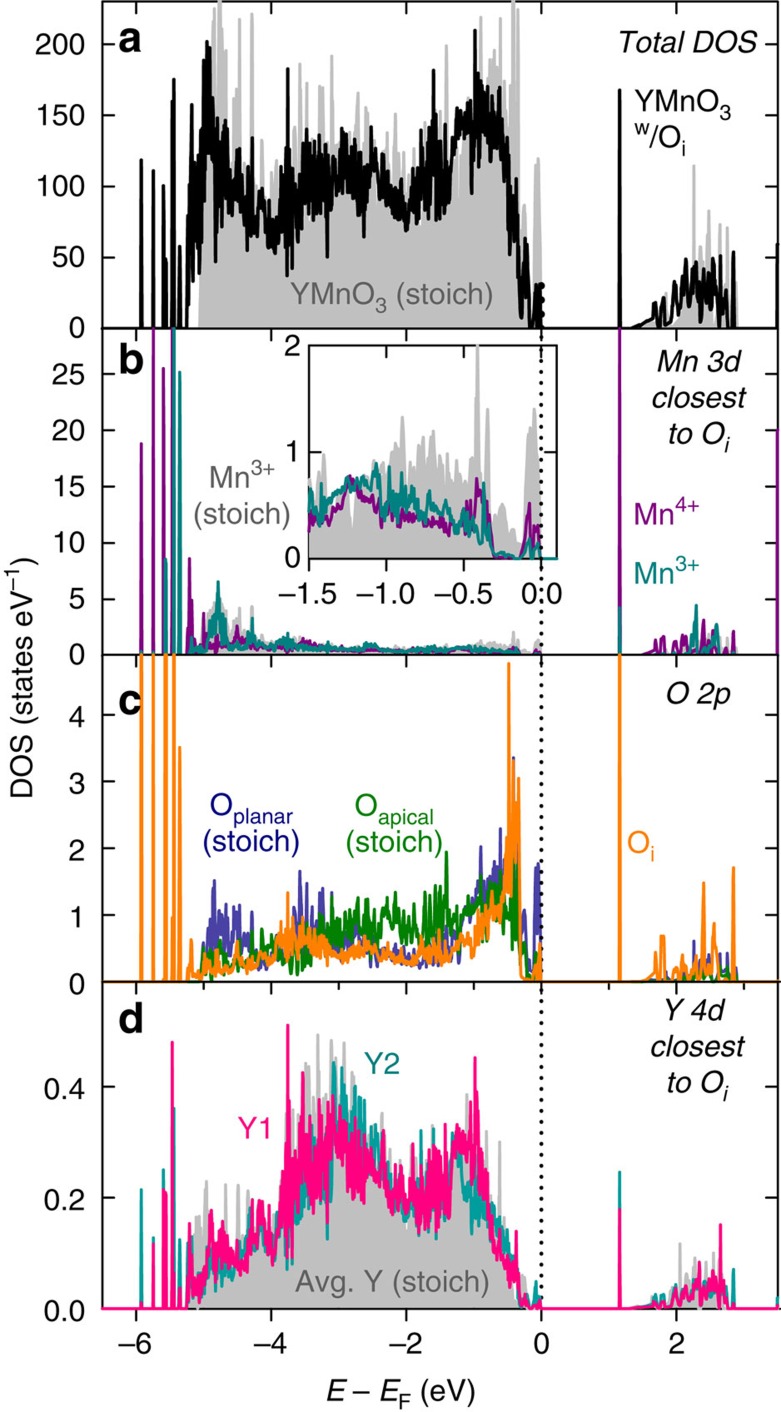
Electronic structure. (**a**) Total electronic density of states (DOS) for a perfect 120 atom YMnO_3_ cell and a 121 atom cell of YMnO_3_ with one O_i_ (YMnO_3.04_). (**b**) Atomic DOS showing the *d* states for Mn^3+^ and Mn^4+^ coordinating O_i_ compared with Mn^3+^ *d* states in a stoichiometric cell. (**c**) Atomic DOS showing the *p* states of O_i_ compared with *p* states for apical and planar oxygens in a stoichiometric cell. (**d**) Atomic DOS showing the *d* states of the closest Y1 and Y2 to O_i_ compared with the average Y *d* states in a stoichiometric cell.

To investigate the ionic mobility of interstitial oxygen, migration paths for O_i_ between two stable positions were investigated by NEB calculations. Simple interstitial migration, with a calculated energy barrier of almost 6 eV (Supplementary Fig. 8a), is not likely to occur. In the interstitialcy mechanism, O_i_ pushes an adjacent planar oxygen into a neighbouring interstitial site, subsequently taking up a regular planar oxygen lattice position itself. The maximum energy along the minimum energy path is found when the distance between O_i_ and planar lattice oxygen goes through a minimum. When O_i_ nudges a regular oxygen at an O3 site, (Fig. 4b) an energy barrier of 0.48 eV was found (Fig. 4c). A similar mechanism where O_i_ nudges a regular oxygen at an O4 site resulted in a higher energy barrier of 0.62 eV (See Supplementary Fig. 8b,c for effect of magnetic order and Hubbard *U*). This is explained by Y1 being closer to the moving O3 (which is a trimerization centre) and O_i_ in path 2 compared with the corresponding distance between Y2 and the moving O4 and O_i_ in path 1 (Fig. 4b) (See Supplementary Fig. 8e and f for illustration of bond lengths). Migration through path 2 will therefore be aided by Y1 bonding to the two moving oxygen atoms, which is supported by the electronic density of states in Fig. 5d. An energy barrier was also estimated from the experimental TGA data by plotting time to oxidation as a function of annealing time in O_2_ atmosphere, as shown in Supplementary Figure 8d. The Arrhenius-type relation gave an energy barrier of 0.55±0.21 eV, in agreement with our DFT calculated values. For comparison, oxygen transport through vacancy diffusion in ABO_3_ perovskites has an energy barrier in the range of 0.5–2.8 eV (ref. 26). See Supplementary Note 4 for more details about the migration barriers.

### Functional properties

We now address the impact of O_i_ on the electronic properties. The introduction of O_i_ between three Mn (Fig. 3a) gives three edge-sharing octahedra. As these octahedra are strongly distorted, the crystal field experienced by the d-electrons of Mn coordination O_i_ does not change significantly compared with the trigonal bipyramidal crystal field in the perfect crystal. The most obvious change in electronic structure, in Fig. 5, caused by O_i_ is the appearance of a non-bonding defect state in the band gap mainly consisting of Mn^4+^ 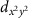 states and O_i_ 2*p*_*x*_ and 2*p*_*y*_ states. A fraction of the occupied Mn *d* states closest to the Fermi energy is lifted above *E*_F_ on inclusion of O_i_ as electron density is donated from Mn 3*d* to O 2*p*. The corresponding binding states are easily seen at the bottom of the valence band. Even though charge transfer is not complete, this can formally be regarded as the oxidation of Mn^3+^ to Mn^4+^, creating holes in the valence band and *p-*type electronic conductivity. The *p*-DOS of interstitial oxygen is very similar to that of planar oxygen, which is also coordinated by only Mn, while it differs significantly from the *p*-DOS of apical oxygen, which is coordinated by both Mn and Y. The highly localized character of the defect state in the band gap is characteristic for the electronic structure of an acceptor doped material, and direct experimental detection of O_i_ in low concentrations, by for example spectroscopic techniques, would be challenging. With increasing O_i_ concentration, the defect state becomes less localized, as expected (See Supplementary Figure 9 for DOS and band structure of a 30 atom unit cell, YMnO_3.16_).

To address the impact of O_i_ on the ferroelectric properties we calculated the spontaneous polarization (*P*_S_) by the Berry phase method to 7.8 μC cm^−2^ for the perfect structure, and 7.2 μC cm^−2^ after inclusion of one O_i_ in a 120 atom supercell. A simple point charge model gave 7.8 and 7.0 μC cm^−2^ for stoichiometric YMnO_3_ and oxidized YMnO_3.04_, respectively. Although the effect of O_i_ on the spontaneous polarization is subtle, the charge compensating holes will raise the electrical conductivity and be detrimental to the macroscopic ferroelectric performance. However, in improper ferroelectrics like YMnO_3_, charged domain walls (DW) display anisotropic conductance due to the accumulation of mobile charge carriers^27^. Interstitial oxygen with formal charge −2 can screen the electrostatic field at head-to-head DWs, while charge compensating holes can screen tail-to-tail DW. Engineering point defect populations at DWs has great potential for tuning the properties of DWs as functional elements for electronics^28^,^29^,^30^,^31^.

To summarize, we have shown that interstitial oxygen is the dominating point defect in YMnO_3_ and the source of *p*-type electronic conductivity at ambient conditions. Interstitial oxygen is an enthalpy stabilized point defect, implying that YMnO_3_ is not only metastable with respect to Y_2_O_3_ and YMn_2_O_5_, in accordance with the phase diagram^32^, it is also metastable with respect to oxidation towards the limiting case of Y_3_Mn_3_O_10_. A bulk YMnO_3_ sample will however not oxidize completely at ambient conditions for kinetic reasons; the diffusion length is too large compared with the relatively low ionic mobility at room temperature. However, at the nanoscale, in thin films and at surfaces, oxidation will occur spontaneously also at ambient conditions. This emphasizes the necessity and potential which lies in controlling the thermal and atmospheric history of hexagonal manganite materials in order to tailor the point defect population, charge carrier concentration and physical properties.

## Methods

### Experimental

Bulk YMnO_3_ powder was prepared by firing pressed pellets of dried and mixed Y_2_O_3_ and MnO_2_ twice for 24 h at 1,300 °C in air. Porous polycrystalline bars with a density of 43% were sintered from phase pure YMnO_3_ powder with 20 wt% carbon black as a pore filler for 2 h at 1,500 °C in air and scanning electron micrographs of the fracture surfaces of the porous bars are given in Supplementary Fig. 10. Porous bars were used to increase the total surface area accessible to oxygen surface exchange. Four-point electrical conductivity measurements were performed on a porous polycrystalline bar in flowing O_2_ and N_2_ (ref. 33). Impedance spectroscopy measurements (Novotherm, Novocontrol Technologies) were conducted within a temperature range from 30 to 350 °C on disc shaped samples using frequencies between 1 Hz and 1 MHz. Before measurement the samples were annealed in flowing oxygen resp. nitrogen for 24 h at 350 °C. Thermogravimetry (TGA) was done with a Netzsch STA 449C Jupiter in flowing N_2_ and O_2_ on nanoparticles with *d*_XRD_=49±4 nm (ref. 17). Seebeck coefficients were measured at 200–500 °C in flowing O_2_ and N_2_ with a ProboStat setup (NorECs AS) on a porous bar in a vertical tubular furnace^34^. More details on the synthesis and characterization can be found in Supplementary Methods.

### Computational

Density functional theory calculations were performed with the VASP^35^,^36^ code and the spin polarized GGA+*U* implementation of Dudarev^37^ with the PBEsol functional^38^. A Hubbard *U* of 5 eV was applied to the Mn 3*d* orbitals in order to reproduce the experimental band gap^39^ and lattice parameters^40^. The projector augmented wave^41^ method was used treating Y(4*s*,4*p*,4*d,*5*s*), Mn(3*s*,3*p*,3*d*,4*s*) and O(2*s*,2*p*) as valence electrons and a plane-wave cutoff energy of 550 eV. Brillouin zone integration was done on a *Γ*-centered 4 × 4 × 2 mesh for the 30 atom unit cell and with the number of *k*-points reduced accordingly for supercells. The non-collinear magnetic structure was approximated by a frustrated collinear antiferromagnetic (F-AFM)^42^ order in most of the calculations, which does not give significantly different results from calculations with the true non-collinear magnetic order. Geometry optimization was done until the forces on the ions were below 0.005 eV Å^−1^ for 121 atom cells and 0.02 for 541 atom cells. The reducing and oxidizing limit for the chemical potential of oxygen varies from −7.1 to −1.9 eV. The defect formation energy of O_i_ was 0.03 eV lower in a 121 atom cell than in a 541 atom cell (See Supplementary Fig. 11). Bader analysis^43^ gave charges of +1.90 and +1.73 for Mn^4+^ and Mn^3+^, respectively. For the nudged elastic band^44^ (NEB) calculations we used five intermediate images for the O_i_ migration path and a Hubbard *U*=5 eV was applied to reproduce the nature of the localized electron holes (comparison with *U*=0 eV can be found in the Supplementary Fig. 8b). A frustrated antiferromagnetic (F-AFM) order was also used for the calculation of the energy barriers. Comparison between A-type AFM and F-AFM magnetic order is given in Supplementary Fig. 8c, and was set so that no spin flipping on the Mn ions occurred during the migration of O_i_ in order to isolate the effect of pure migration. The ferroelectric polarization was calculated with the Berry phase method^45^. VESTA was used for visualizing structures^46^.

### Data availability

The data that support the findings of this study are available from the authors on reasonable request.

## Additional information

**How to cite this article:** Skjærvø, S. H. *et al*. Interstitial oxygen as a source of *p*-type conductivity in hexagonal manganites. *Nat. Commun.* **7,** 13745 doi: 10.1038/ncomms13745 (2016).

**Publisher's note**: Springer Nature remains neutral with regard to jurisdictional claims in published maps and institutional affiliations.

## Supplementary Material

Supplementary Information Supplementary Figures, Supplementary Notes, Supplementary Methods and Supplementary References.
