# Supplementary material for: Interstitial oxygen as a source of p-type conductivity in hexagonal manganites
Source: Nat Commun. 2016 Dec 7;7:13745. doi: 10.1038/ncomms13745 (PMC5150987; doi:10.1038/ncomms13745)
Supplement: Supplementary Information — Supplementary Figures, Supplementary Notes, Supplementary Methods and Supplementary References. [file ncomms13745-s1.pdf]

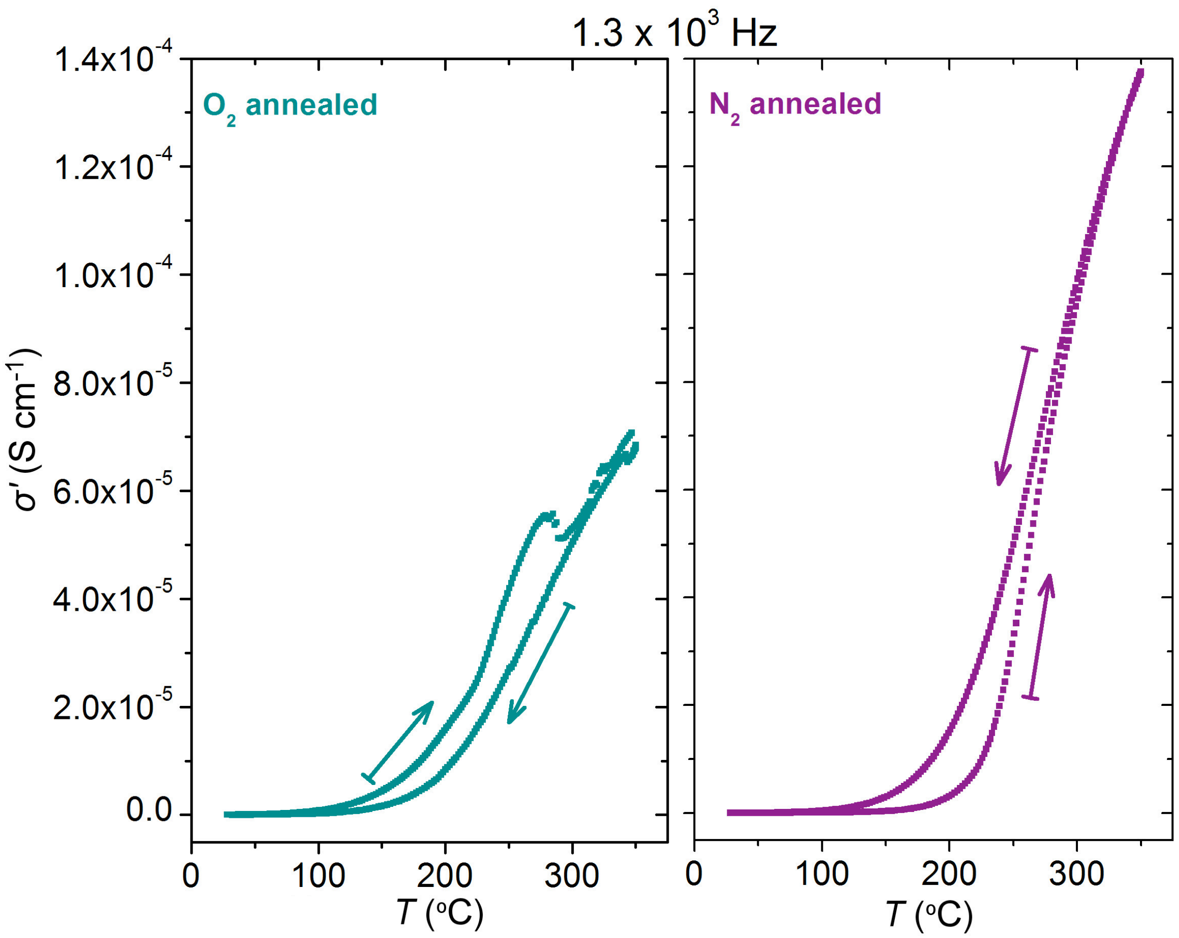


**Supplementary Figure 1: AC conductivity**, $\sigma'$, measured on an $YMnO_3$ polycrystal between room temperature and 350 °C in air. The measurement frequencies ranged from 1 Hz to 1 MHz. The results for $f$ = 1.3 kHz are shown for illustration. The left hand panel depicts the result for a sample annealed in flowing $O_2$, the right hand panel for a sample annealed in flowing $N_2$. In both cases the heat treatment was conducted for 24 hours at 350 °C prior to measurement.

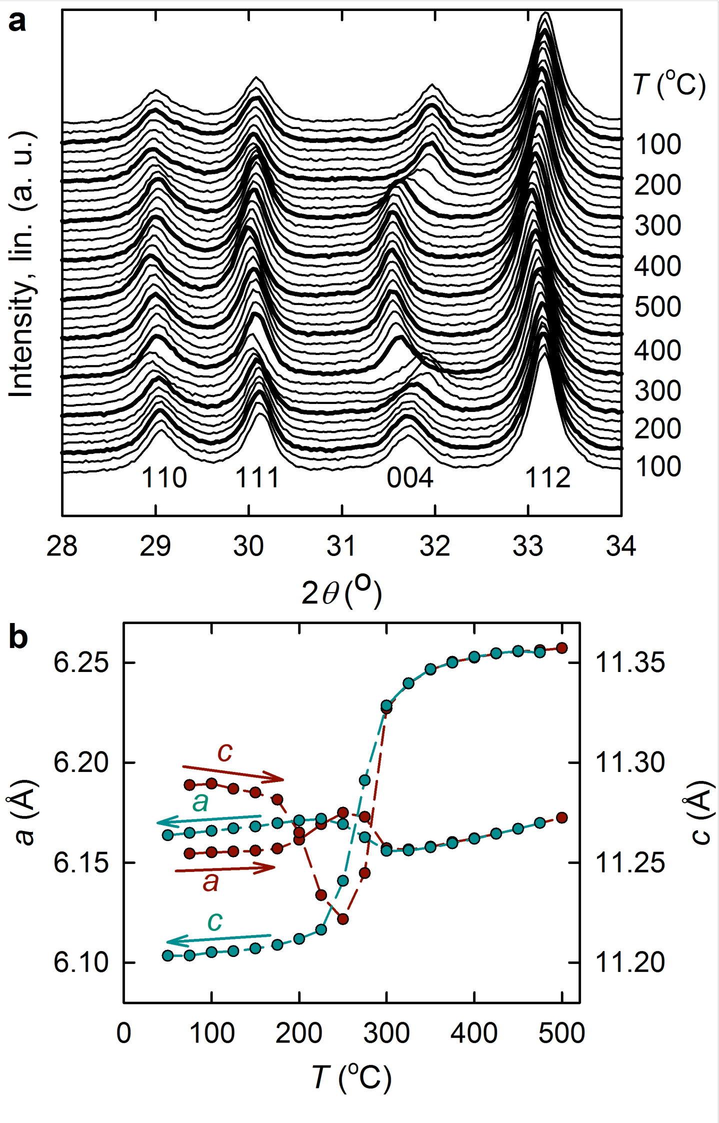


**Supplementary Figure 2: a) High temperature X-ray diffraction** (HTXRD) of nanocrystalline $YMnO_3$ upon one heating and cooling cycle in $O_2$ between 50 and 500 °C plotted on linear scale, and **b)** lattice parameters, *a* and *c* extracted from the measurements of two heating and cooling cycles, showing that interstitial oxygen is incorporated into the lattice, causing anisotropic chemical expansion. No evidence of secondary phases could be seen for any of the temperatures in the $2\theta$ range between 26 and 72°.

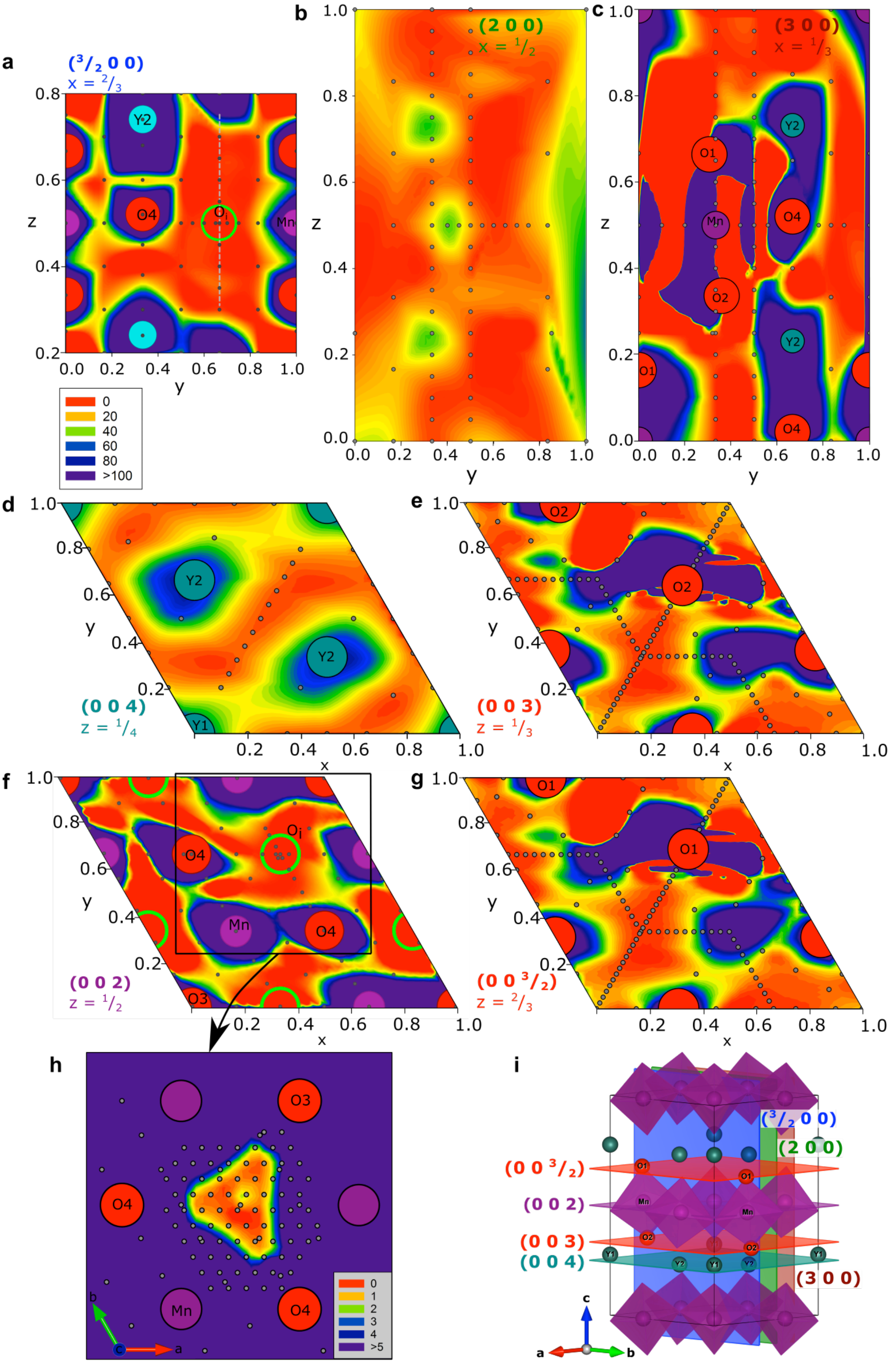


**Supplementary Figure 3: a-h) Potential energy surface (PES) mapping** of several planes in a 2x2x1 supercell (excerpt of only the unit cell) of $YMnO_3$. **i**) Unit cell showing the crystallographic planes that were mapped.

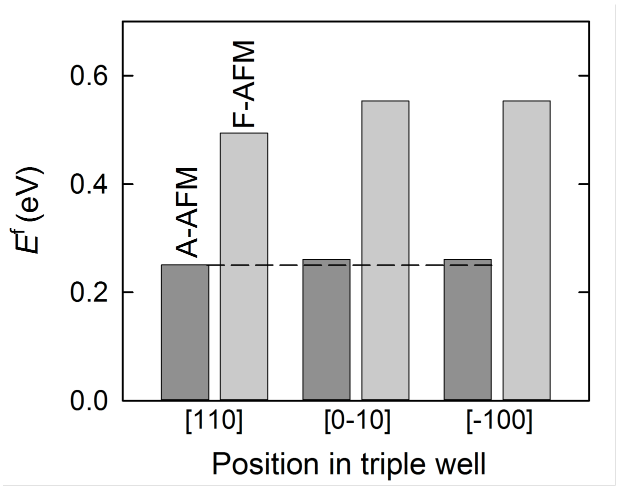


**Supplementary Figure 4: Energetic asymmetry of the $O_i$ triple well.** Defect formation energies of $O_i$ in the three triple well positions calculated with the F-AFM and A-AFM order, comparing contributions from magnetism and structural trimerization.

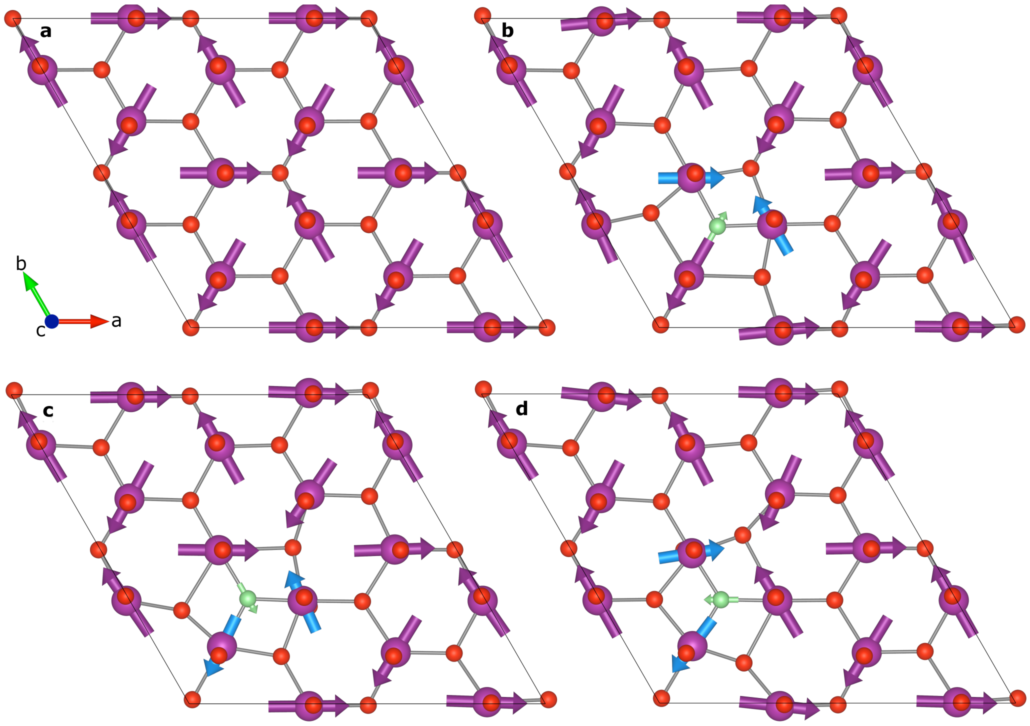

**Supplementary Figure 5: Effect of $O_i$ on true non-collinear magnetic order.** Static calculations with Hubbard $U$ = 5 eV and the true non-collinear order of structurally relaxed cells with $O_i$. **a)** stoichiometric cell, and **b-d)** defective cells with $O_i$ relaxed in one of the three triple well positions.

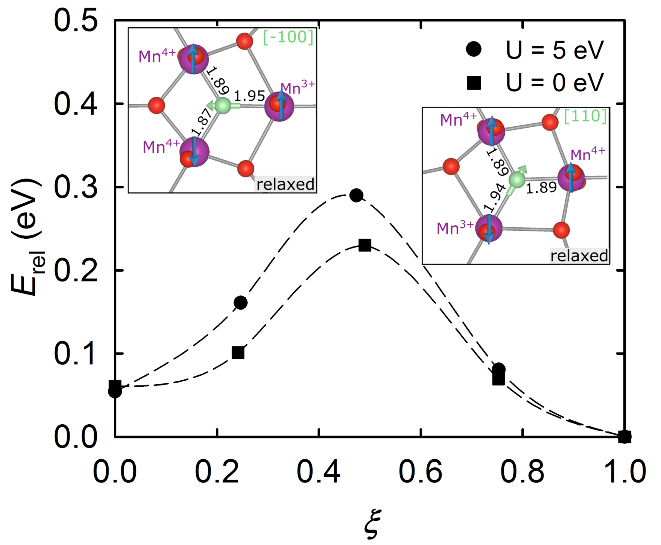


**Supplementary Figure 6: Polaron hopping barrier.** Energy barrier as a function of the relative reaction coordinate for moving $O_i$ and the charge compensating electron holes from one site in the triple well to another. The blue arrows show the collinear frustrated antiferromagnetic (F-AFM) order used in the calculations. The apparent energy difference in the start and end configuration is caused by the energy asymmetry of the triple well.

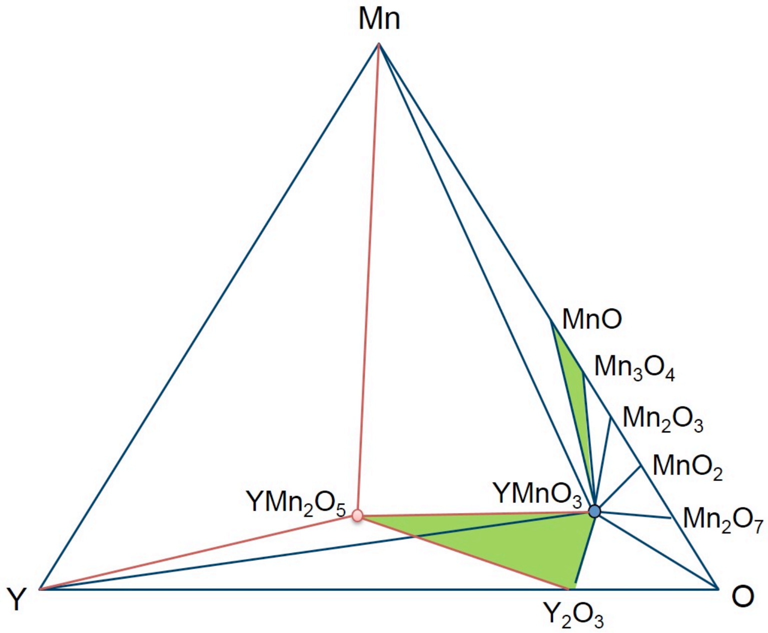


**Supplementary Figure 7: Phase equilibrium triangle** showing the phase equilibria which determine the phase stability of h-$YMnO_3$. The green areas show the phase equilibria which were used to define the stability region of $YMnO_3$.

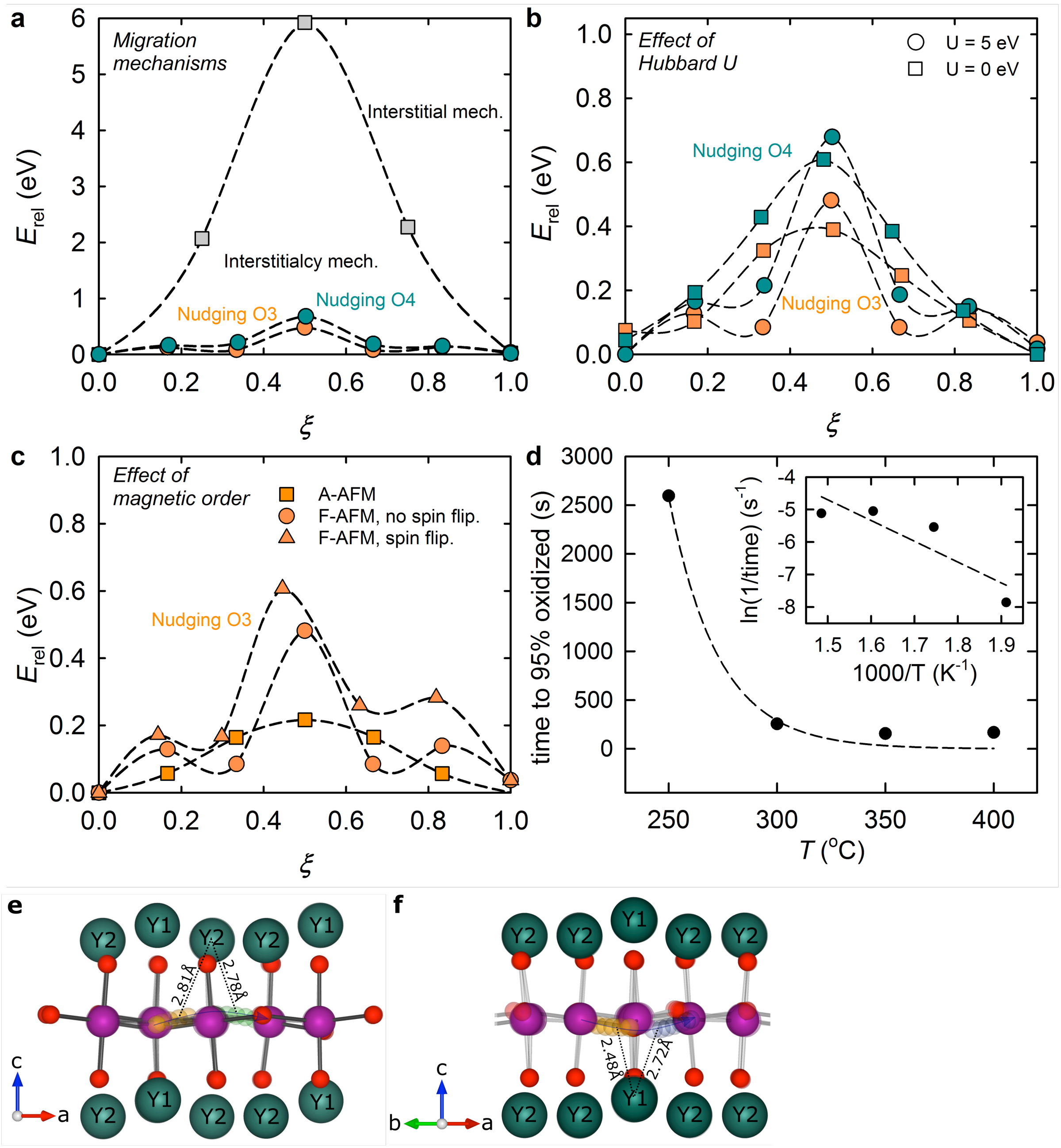


**Supplementary Figure 8: Migration barriers for $O_i$ a)** Energy barriers for migration by interstitialcy mechanism (nudging O3 or O4) compared to an interstitial mechanism as a function of relative reaction coordinate, *ξ*. **b)** Calculated energy barriers for migration of interstitial oxygen interstitialcy mechanism comparing the effect of on-site Coulomb repulsion by Hubbard *U* = 0 eV and *U* = 5 eV. **c)** Calculated energy barriers for interstitialcy migration through O3 comparing the effect of the magnetic orders frustrated antiferromagnetic (F-AFM) with or without spin flipping and A-type antiferromagnetic (A-AFM). **d)** Time to 95% oxidation of nanoparticles as a function of temperature from thermogravimetric isotherms. The inset shows the data in an Arrhenius-type plot used to extract energy barrier for diffusion. **e)** Bonds between Y2 and the moving oxygens in the middle of path 2. **f)** Bonds between Y1 and the moving oxygens in the middle of path 1.

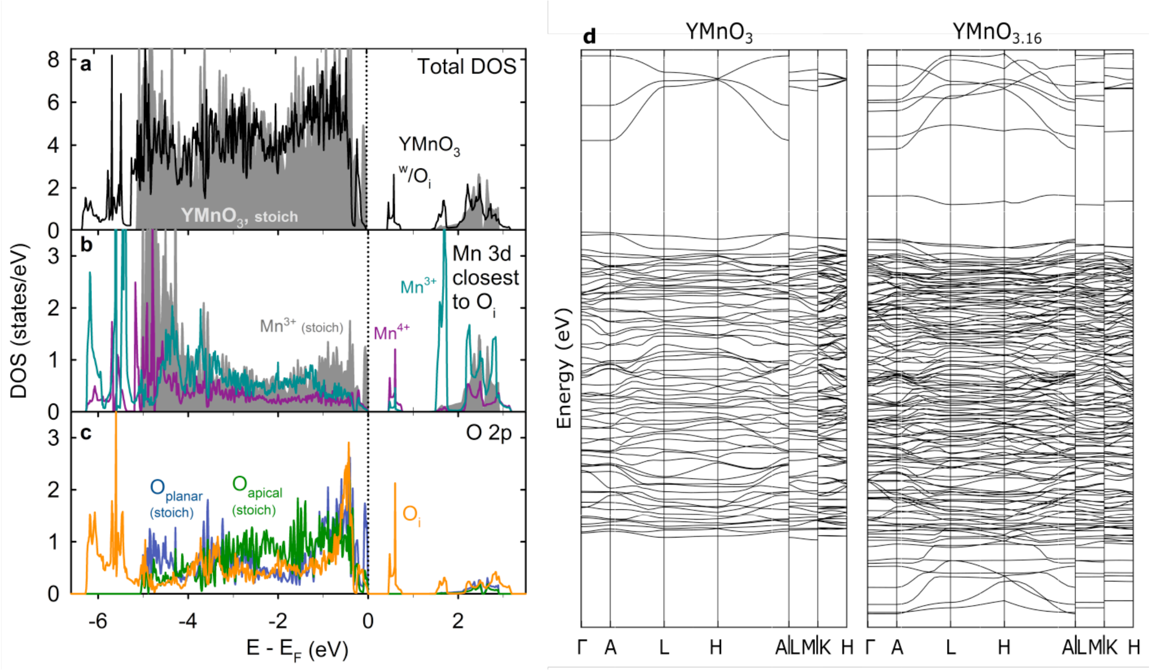


**Supplementary Figure 9: Electronic structure. a**, Total electronic density of states (DOS) per formula unit for perfect $YMnO_3$ and a 30 atom cell of $YMnO_3$ with one $O_i$ ($YMnO_{3.16}$). **b**, Atomic DOS showing the d states for $Mn^{3+}$ and $Mn^{4+}$ coordinating $O_i$ compared to $Mn^{3+}$ d states in a stoichiometric cell. **c**, Atomic DOS showing the p states of $O_i$ compared to p states for apical and planar oxygens in a stoichiometric cell. **d**, Electronic band structure of a stoichiometric 30 atom unit cell compared to a 30 atom unit cell with one $O_i$.

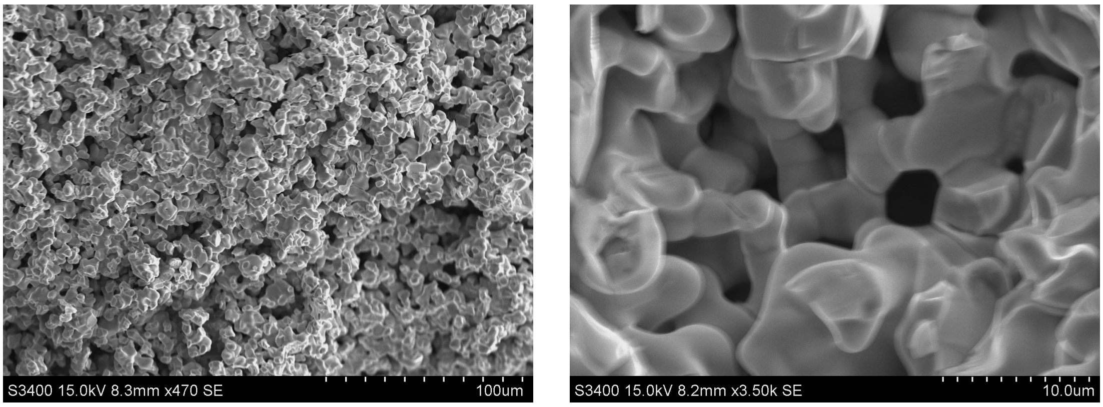


**Supplementary Figure 10: SEM images** of the fracture surface of a porous polycrystalline $YMnO_3$ bar.

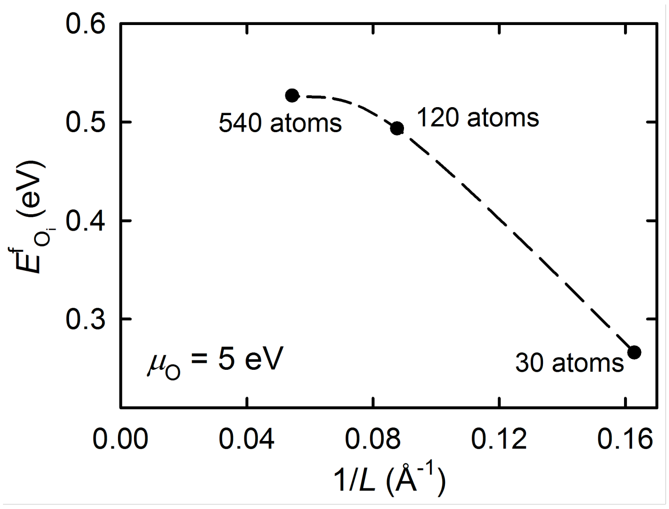


**Supplementary Figure 11: Effect of supercell size on defect formation energy.** Defect formation energy as a function of defect-defect distance illustrating the effect of supercell size.

## **Supplementary Note 1:** Mapping the energy landscape of interstitial oxygen

Potential energy surfaces (PES) were determined by mapping the energy landscape of the $O_i$ position in several lattice planes by static DFT calculations. Seven different lattice planes were mapped, as shown in Supplementary Figure 3 a-g along with a unit cell with the corresponding lattice planes, panel i. The most stable positions of $O_i$ was found to be between three Mn in the Mn-O planes at $z = 0$ and $^1/_2$, which, due to symmetry, results in 6 equivalent possible sites for $O_i$ in the $P6_3cm$ unit cell, ($^1/_3$, $^1/_3$, 0), ($^2/_3$, 0, 0), (0, $^2/_3$, 0), ($^2/_3$, $^2/_3$, $^1/_2$), ($^1/_3$, 0, $^1/_2$) and (0, $^1/_3$, $^1/_2$), as illustrated by green circles in the (002) plane in the figure. An excerpt around the most stable position in the (2 0 0) plane was mapped more thoroughly, panel h, and revealed an asymmetric triangular energy landscape. This shows how forces between the $O_i$ and the three $Mn^{3+}$ eventually leads to a triple well which $O_i$ can relax into and oxidize two of the $Mn^{3+}$ to $Mn^{4+}$.

The six equivalent positions for $O_i$ do not correspond to high-symmetry positions in the $P6_3cm$ space group and the presence of a single $O_i$, without structural relaxation, reduces the symmetry to monoclinic Cm.[1] A fully oxidized structure $YMnO_{3.33}$ with one $O_i$ in each Mn-O layer lowers the symmetry to Cc or $Cmc2_1$[1] depending on which sites the two $O_i$ occupy.

## **Supplementary Note 2:** Energetic asymmetry of the $O_i$ triple well

Depending on which two $Mn^{3+}$ are oxidized to $Mn^{4+}$ by $O_i$, the $O_i$ can relax into one of three positions. The origin of the energy asymmetry of this triple well is caused by the characteristic trimerization pattern of the structure and magnetic order. As seen in Supplementary Figure 4, the $O_i$ favours to relax in the [110] direction, which is towards a trimerization center, an O3 atom, towards which the surrounding trigonal bipyramids are all tilting towards or away from. This position results in an Y1-$O_i$ bond length which is shorter than the corresponding Y2-$O_i$ bond length in the two other positions. The magnetic order follows the trimerization pattern and emphasizes the energy asymmetry. In most calculations, the non-collinear magnetic structure[2] was approximated by a synthetic collinear frustrated anti-ferromagnetic (F-AFM) ordering, and this simplification gives an additional unwanted contribution to the asymmetry. The F-AFM order is believed to create stronger electron repulsions than the true non-collinear spin and creates an additional asymmetry in the structure. If the F-AFM order is not set in a way which preserves the structural symmetry of the triple well, additional local minima can trap $O_i$ and the structure distorts unphysically. The F-AFM order should be set so the most favoured triple well position is in the direction of two Mn with equal spin. Setting an A-type antiferromagnetic (A-AFM) order also results in the same triple well asymmetry, but not as pronounced. It is believed to give even smaller electronic repulsion than the true non-collinear magnetic order. Attempts of calculating the defect formation energies in the triple well with non-collinear magnetic order failed due to VASP not being able to reproduce the true non-collinear magnetic ground state. In conclusion, the most favoured $O_i$ triple well position causes minimal and more symmetric distortion of the surrounding bipyramids.

**Supplementary Note 3:** Polaron hopping barrier

Nudged elastic band calculations between the different sites in the triple well show that the energy barrier for displacing the $O_i$ between different Mn pairs is 0.29 eV, as shown in Supplementary Figure 6. The barrier is found to be mainly determined by electron transfer between $Mn^{3+}/Mn^{4+}$, including spin flipping because of the synthetic collinear frustrated antiferromagnetic (F-AFM) order, and can thus be seen as the barrier for polaron hopping. The high energy barrier suggests that the electron holes will be localized in the whole temperature stability range of $O_i$ in the $P6_3cm$ structure. When the on-site Coulomb potential is reduced by not setting a Hubbard $U$ ($U$ = 0 eV), the energy barrier is reduced by 0.06 eV.

**Supplementary Note 4:** Migration of interstial oxygen

The interstitial oxygen can migrate through the structure by an interstitialcy mechanism nudging a lattice oxygen O3, with an energy barrier of 0.48 eV as shown in Supplementary Figure 8 a and b. The $O_i$ can also migrate through a similar mechanism nudging a lattice oxygen O4 instead, but this gives a higher migration barrier of 0.62 eV. We expect all the calculated barriers to be slightly higher than in reality due to the synthetic collinear magnetic (F-AFM) order which can give larger repulsion between the electrons than in the paramagnetic state. Diffusion through this interstitialcy mechanism is highly favoured over a pure interstitial mechanism where the $O_i$ migrates alone out of the Mn-O plane, shown in Supplementary Figure 8 a. The structural effects of the $O_i$ are well screened, mainly due to the high structural flexibility of the trigonal bipyramids. Since they are not rigid polyhedra, the introduction of an $O_i$ is structurally screened by changing the bond angles of the polyhedra, rather than rotating the polyhedra. As the $O_i$ passes by the Mn atoms in the migration path, the tilting of the nearest trigonal bipyramids is reduced.

The migration path of $O_i$ was analyzed using the nudged elastic band method as implemented in VASP with five intermediate images[3]. A Hubbard $U$ = 5 eV was used because it described the nature of the localized electron holes better and thereby also capturing the polaron hopping as one of the main contributions to the energy barrier. Test calculations with $U$ = 0 eV gave on average 0.07 eV lower energy barriers, as seen in Supplementary Figure 8 b, but the calculations suffered from slower convergence due to charge sloshing and often less physical results.

Our calculations show that a frustrated antiferromagnetic (F-AFM) order gives energy barriers which are in accordance with experimental energy barriers, both for migration of $O_i$ and polaron hopping. Setting an A-type antiferromagnetic (AFM) order gave on average 0.27 eV lower energy barrier. This magnetic order gives equal spin direction for all the Mn within the same layer and a much smaller bandgap, which is believed to be the cause of the lower barrier. The F-AFM order was set in such a way that spin flipping on Mn ions did not occur during migration of $O_i$, in order to isolate the effect of only migration. Test calculations where spin flipping occurred during migration gave 0.14 eV higher energy barrier, Supplementary Figure 8 c.

The migration energy barrier was also obtained from the thermogravimetric isothermal data in $O_2$, by plotting the time to 95% oxidation as a function of temperature shown in Supplementary Figure 8 d. Plotting $t^{-1}$ as a function of the inverse temperature gave a linear Arrhenius-type behaviour following $1/t = Ae^{-E_a/kT}$. The slope of the line gave an energy barrier of $E_a = 0.55 \pm 0.21$ eV, which is comparable to the calculated energy barrier of 0.48 eV.

## Supplementary Methods

### Thermodynamic stability region for h-$YMnO_3$

The stability of a compound with respect to its related binary or ternary compounds is dependent on the chemical potential of oxygen in the surroundings. For instance, at reducing conditions, $YMnO_3$ can decompose to MnO (s), $Y_2O_3$ (s) and $O_2$ (g), but the particular chemical potential of oxygen when this occurs, is not so easy to calculate directly, as the chemical potential of a gaseous species is not well defined at 0 K. Instead we calculate the ground state energies of all the solid compounds in the related chemical reaction, and extract $\mu_O$ as the only unknown. All the phase equilibria which determine the stability region of h-$YMnO_3$ are presented in Supplementary Figure 7. Each area of the triangle corresponds to values of the chemical potentials for all three elements, $\mu_Y$, $\mu_{Mn}$ and $\mu_O$. Calculations of $\mu_O$ from all the areas provide the range in which $\mu_O$ can vary. The oxidizing and reducing limits were found to be determined by the chemical reactions given below. Chemical equilibria with elemental Y and Mn are omitted.

Reducing conditions:

$$3\,\mathrm{MnO} + \frac{1}{2}\mathrm{O_2(g)} \rightleftarrows \mathrm{Mn_3O_4} \qquad \mu_O = \mu_{\mathrm{Mn_3O_4}} - 3\mu_{\mathrm{MnO}} = \text{-7.1 eV}$$

Oxidizing conditions:

$$4\,\mathrm{YMnO_3} + \frac{1}{2}\mathrm{O_2(g)} \rightleftarrows \mathrm{Y_2O_3} + 2\,\mathrm{YMn_2O_5} \qquad \mu_O = \mu_{\mathrm{Y_2O_3}} + 2\mu_{\mathrm{YMn_2O_5}} - 4\mu_{\mathrm{YMnO_3}} = \text{-1.9 eV}$$

### Materials synthesis

$YMnO_3$ powder was prepared by solid state reaction between $Y_2O_3$ (Alfa Aesar >99.9%) and $MnO_2$ (Sigma-Aldrich, >99%). The powders were dried overnight at 500 °C before weighing and mixed in ethanol in a mortar. Uniaxially pressed pellets were fired twice for 24 hours at 1300 °C in air with intermediate grinding and heating and cooling rates of 200 °C $h^{-1}$. Phase purity was confirmed by XRD between each heating step. Powder for sintering porous polycrystalline bars for electrical conductivity and Seebeck measurements were prepared by mixing phase pure $YMnO_3$ powder with 20 wt% carbon black (Merck) followed by ball milling for 4 hours in ethanol with yttrium stabilized zirconia milling balls. Green bodies were isostatically cold pressed at 200 MPa in a Painan Autoclave Engineers CIP and 1 wt% binder of cellulose acetate (Aldrich) dissolved in acetone was added to improve the green body strength. The porous polycrystalline bars were sintered for 2 hours in air at 1500 °C after a binder burn-off step at 435 °C and polished to dimensions of 15 x 5 x 5 mm. The density of the bar was measured by the Archimedes method[4] to be 43 %. A representative fracture surface of a polycrystalline porous bar is shown in Supplementary Figure 10. Scanning electron microscopy of the fracture surface was done with a Hitachi S-3400N.

## Materials characterization

Four-point electrical conductivity measurements in $O_2$ and $N_2$ (Yara Praxair, 5.0) atmospheres were performed with a home-built instrument described elsewhere[5]. Platinum paste was applied to the interface between the platinum electrode wires and the sample and heated *in situ* to 1000 °C to ensure good electrical contact. The conductivity $\sigma$ was calculated from the equation $\sigma = {Id}/{AV}$ where $I$ is the current, $d$ is the distance between the electrodes, $A$ is the sample cross section area and $V$ is the measured voltage drop between the electrodes. *In situ* heating and cooling was done with 1 °C min$^{-1}$. Thermogravimetric measurements were done on $YMnO_3$ nanoparticles[6] with $d_{XRD}$ = 49±4 nm used for electrical conductivity measurements with a Netzsch STA 449C Jupiter in flowing $N_2$ and $O_2$ (Yara Praxair, 5.0). For both the conductivity and thermogravimetric measurements, the sealed system for conductivity measurements could not be evacuated between the changes of atmosphere. Thus, the exact $pO_2$ in $N_2$ atmosphere is not known exactly, but estimated to be in the range of $10^{-4}$-$10^{-5}$. Seebeck-coefficients ($Q$) were measured at 400, 350, 300, 250 and 200 ºC in $O_2$ (Yara Praxair, 5.0) using a ProboStat™ setup (NorECs AS) on a vertically aligned polycrystalline porous bar (radius 3 mm, length 12 mm) in tubular furnace. Two S-type thermocouples (20-25 ºC) were used to measure the temperature gradient and the voltage across the sample was measured with Pt-electrodes.

Impedance spectroscopy measurements (Alpha-A analyzer combined with Novotherm furnace, Novocontrol Technologies) were conducted on disc shaped samples with sputtered gold electrodes. Samples were subjected to two temperature cycles from 30-350 ºC with a heating rate of 2 ºC min$^{-1}$. Frequency sweeps covering a range from 1 Hz and 1 MHz were conducted continuously during the temperature cycles. Prior to measurement, the samples were annealed in flowing oxygen resp. nitrogen for 24 hours at 350 ºC .

For the purpose of confirming oxygen incorporation into the lattice in the relevant temperature interval we have instead performed high temperature X-ray diffraction with in-situ change of atmosphere on $YMnO_3$ nanoparticles with an average size of 25 nm. The measurements were performed in-house with a Bruker D8 Advance diffractometer with Cu K$\alpha$ radiation and 20 second collection time per degree from 26 to 72$^o$ 2$\theta$, mapping the evolution of the 004 and 112 Bragg reflections. The lattice parameters $a$ and $c$ upon heating and cooling in $O_2$ atmosphere were extracted by fitting the $d$-spacing of the 004 and 112 Bragg reflections to Lorentzian peak shapes.

## Computational details

The effects of interstitial oxygen were modeled using 2x2x1 unit cells of 121 atoms and 3x3x2 unit cells of 541 atoms with the number of $k$-points reduced accordingly. A 2x2x1 supercell was found to be sufficiently large as it provided a small defect-defect interactions compared to the computational cost, as seen in Supplementary Figure 11 where the defect formation energy for neutral cells is calculated for three cell sizes. No significant change in ferroelectric polarization was found for increasing the cell size from 121 to 541 atoms.

Charge neutral cells were used for the simulations due to the relatively high concentration of $O_i$ in a 120 atom cell, and because, unlike conventional semiconductors, high defect concentrations are observed experimentally. Simulations with cells of charge -1 and -2, where electrons were artificially added to avoid charge compensating oxidation of $Mn^{3+}$ to $Mn^{4+}$, gave the same results as neutral cells with respect to the position of $O_i$.

### Supplementary References

1. Stokes H. T. & Hatch D. M. FINDSYM: program for identifying the space-group symmetry of a crystal. *J. Appl. Cryst.* **38**, 237-238 (2005). 10.1107/S0021889804031528

2. Howard C. J., Campbell B. J., Stokes H. T., Carpenter M. A. & Thomson R. I. Crystal and magnetic structures of hexagonal $YMnO_3$. *Acta Crystallogr. B* **69**, 534-540 (2013). 10.1107/S205251921302993X

3. Henkelman G., Uberuaga B. P. & Jonsson H. A Climbing image nudged elastic band method for finding saddle points and minimum energy paths. *J. Chem. Phys.* **113**, 9901-9904 (2000). 10.1063/1.1329672

4. ISO 5017:1998 *Dense shaped refractory products - Determination of bulk density, apparent porosity and true porosity*.

5. Selbach S. M., Tybell T., Einarsrud M.-A. & Grande T. High-temperature semiconducting cubic phase of $BiFe_{0.7}Mn_{0.3}O_{3+\delta}$. *Phys. Rev. B* **79**, 214113 (2009). 10.1103/PhysRevB.79.214113

6. Bergum K., Okamoto, H, Fjellvåg H., Grande, T, Einarsrud M.-A. & Selbach, S. M. Synthesis, structure and magnetic properties of nanocrystalline $YMnO_3$. *Dalton Trans.* **40**, 7583-7589 (2011). 10.1039/c1dt10536a
